# Initial end-to-end testing of the ExacTrac dynamic deep inspiration breath hold workflow using a breath hold breast phantom

**DOI:** 10.1007/s13246-023-01291-y

**Published:** 2023-06-22

**Authors:** Simon K. Goodall, Peter L. Rampant

**Affiliations:** 1 School of Physics, Mathematics, and Computing, Faculty of Engineering and Mathematical Sciences, University of Western Australia, Crawley, WA 6009 Australia; 2 GenesisCare, 24 Salvado Road, Wembley, WA 6014 Australia

**Keywords:** Deep inspiration breath hold, ExacTrac Dynamic, Surface guided Radiotherapy, Stereoscopic breast imagining

## Abstract

ExacTrac Dynamic (ETD) provides a Deep Inspiration Breath Hold (DIBH) workflow for breast patients. Stereoscopic x-ray imaging combined with optical and thermal mapping allows localisation against simulation imaging, alongside surface guided breath hold monitoring. This work aimed to determine appropriate imaging parameters, the optimal Hounsfield Unit (HU) threshold for patient contour generation and workflow evaluation via end-to-end (E2E) positioning using a custom breast DIBH phantom. After localisation via existing Image Guidance (IG), stereoscopic imaging was performed with a range of parameters to determine best agreement. Similarly, residual errors in prepositioning were minimised using a range of HU threshold contours. E2E positioning was completed for clinical workflows allowing residual isocentre position error measurement and existing IG comparison. Parameters of 60 kV and 25mAs were determined appropriate for patient imaging and HU thresholds between −600 HU and −200 HU enabled adequate prepositioning. The average and standard deviation in residual isocentre position error was 1.0 ± 0.9 mm, 0.4 ± 1.0 mm and 0.1 ± 0.5 mm in the lateral, longitudinal and vertical directions, respectively. Errors measured using existing IG were −0.6 ± 1.1 mm, 0.5 ± 0.7 mm and 0.2 ± 0.4 mm in the lateral, longitudinal and vertical directions, and 0.0 ± 1.0^o^, 0.5 ± 1.7^o^ and −0.8 ± 1.8^o^ for pitch roll and yaw. The use of bone weighted matching increased residual error, while simulated reduction of DIBH volume maintained isocentre positioning accuracy despite anatomical changes. This initial testing indicated suitability for clinical implementation during DIBH breast treatments.

## Introduction

Deep Inspiration Breath Hold (DIBH) has shown clinical benefits to patients undertaking a course of left breast radiotherapy [1–4]. A deep inhalation of air can increase the distance between heart and breast tissue allowing lower heart doses to be achieved during treatment planning [1–4]. Additionally, the respiratory motion of the breast is minimised resulting in reduced positional uncertainty when compared to ungated treatments.

To enable high quality DIBH treatment delivery, a patient must be guided into a reproducible position on multiple days of treatment following an initial simulation. The DIBH system should be able to verify the treatment target is positioned correctly relative to the isocentre of the linear accelerator (linac) and that positioning has been achieved via elevation of target relative to the organ at risk (OAR). Surface Guidance (SG) systems have shown promise in breast DIBH due to their ability to assess the patient external surface surrounding the treatment area [5–9]. The superficial treatment volume ensures the patient external surface provides a good surrogate for the treatment volume when the chest region is monitored [8, 10].

Alderliesten et al. compared positioning errors reported by Cone Beam Computed Tomography (CBCT) with those reported by the AlignRT (Align RT, Vision RT Ltd, London, UK) system for 20 patients [11]. They showed agreement with systematic errors less than 3 mm and random errors less than 2 mm. The 95% limits of agreement were less than 5 mm when considering the left breast alone. Orthogonal MV/kV image pairs were used by Nankali et al. to assess the accuracy of chest wall positioning by the AlignRT system. They determined PTV margins of up to 3.5 mm were required to account for the observed treatment errors [12]. Crop et al. showed that the Catalyst (C-Rad, Uppsala, SE) system in combination with the Halcyon (Varian Medical Systems, Palo Alto, USA) could achieve sub mm positioning for a non-deforming phantom [13]. All these results were within the TG302 recommended values for DIBH breast treatments of 3-5 mm and 2-3^O^ [5].

The ExacTrac Dynamic (ETD) system (Brainlab AG, Munich, Germany) consists of an optical structured light scanning (SLS) and thermal surface tracking system coupled with a stereoscopic x-ray imaging system. Full descriptions of the platform and calibration procedures have been published previously [14, 15]. With ETD software version 1.1 a breast DIBH treatment workflow utilising both SG and x-ray imaging was released. Before such workflows are used in clinical practice, their performance should be evaluated to guide clinicians and to allow optimal performance to be achieved [5, 9].

Published guidelines describe testing which should be completed to evaluate SGRT systems [5, 9]. A range of these tests are covered during the Brainlab Customer Acceptance Testing (CAT) to provide confidence in the system installation and performance. The SLS can be used in isolation to position a patient ready for pre-treatment image guidance (prepositioning) and has been shown to perform this process to sub-millimetre accuracy for a static cranial phantom [16]. During treatment the SLS and thermal system work in combination to detect relative motion. Chow et al. and Da Silva et al. showed the optical and thermal system was able to detect motion with submillimetre accuracy in stereotactic radiotherapy centred investigations [14, 15].

CBCT is routinely used for image guided radiotherapy (IGRT) due to the ability to visualise volumetric information. These images can be used to assess internal anatomy including the target location and spacing between breast tissue and the heart. The ETD stereoscopic imaging however provides faster image capture and a lower imaging dose [15, 17, 18]. When correlating the patient external surface obtained by the SG systems to x-ray imaging, short exposure times are optimal as they allow simultaneous image capture with a comparable imaging duration. Reduced imaging dose also reduces the out of field OAR doses such as those of the heart. Planar imaging has previously been shown to offer sufficient information for clinical set-up of tissue-based targets [12, 19].

Following an initial evaluation of the system, end to end (E2E) testing should be completed in conditions similar to those expected in the clinical workflow [5, 9]. This can verify the workflow as a whole, provide training for the clinical team and help to mitigate unexpected errors or challenges before a patient treatment [20]. E2E testing results of the ExacTrac and ETD systems have been published for stereotactic workflows [15, 17], but not for DIBH workflows with soft tissue targets in the breast.

This work aimed to determine appropriate x-ray exposure parameters for stereoscopic breast imaging and the optimal Hounsfield Unit (HU) threshold to be used for thresholding during creation of a patient surface contour within the Treatment Planning System (TPS). It then aimed to evaluate the clinical workflow via E2E testing of expected clinical scenarios to determine the composite residual error in positioning of a phantom.

## Methods

### ETD workflow

The ETD workflow requires the patient to be CT simulated during free breathing (FB) and breath hold (BH). External patient contours are created for both scans and exported to ETD. On the day of treatment, the patient, whilst free breathing, is prepositioned ready for IGRT, using the SLS system alone considering the entire patient external contour. A tracking point is defined by the treatment team approximately at the sternum and a surface area of interest is generated covering the breast and immediate surrounding tissue. ETD calculates the difference in height between the FB and BH contours at the location of the tracking point which defines the BH level of the day.

The patient is subsequently guided into BH. When the tracking point is elevated by the BH level of the day, SLS, thermal and x-ray images are captured simultaneously. Patient positing can be corrected based on x-ray image fusion and any corrections are equally applied to the captured SLS and thermal images of the tracking area. This provides a reference surface of the day, based on stereoscopic x-ray imaging referenced to the CT used in the TPS.

Treatment is delivered during periods where both the tracking point has achieved the correct elevation, and the SLS and thermal images show the patient surface to be positioned correctly. For this work the default ± 2 mm tolerance was applied to both the tracking point height and the surface area.

### Phantom design and validation

**Fig. 1:**
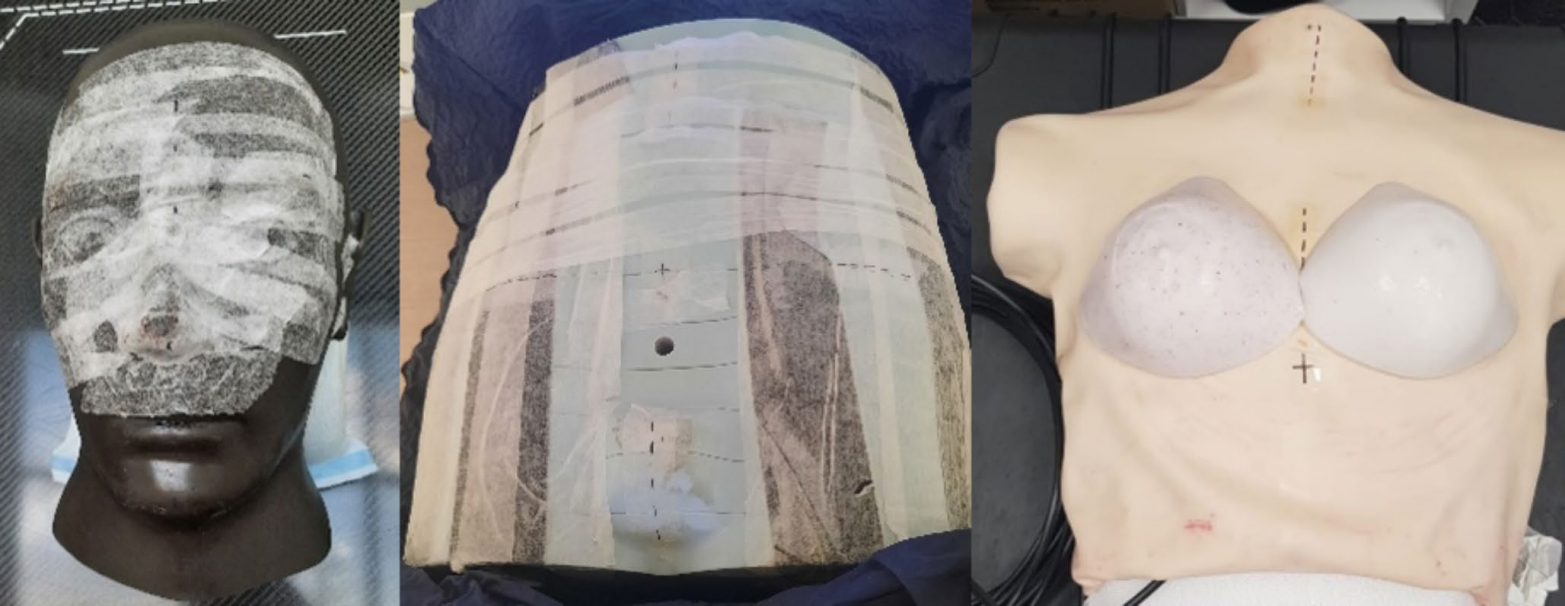
Phantoms used from left to right, head phantom, pelvis (Atom) phantom and modified breast phantom

The phantoms used in this work are shown in Fig. 1. The ExacTrac head phantom (Brainlab AG, Munich, Germany) was used, with and without white tape, a CIRS 701 Atom phantom (Computerized Imaging Reference Systems Tissue Simulation Technology, Norfolk, USA) and a Dynamic Breathing Phantom (Radiology Support Devices Inc, Long Beach, California, USA), modified inhouse for use as a breast phantom with breath hold capability.

The head and pelvis phantom were used for optimal external contour threshold testing only, while the breast phantom was used for all other testing. To modify the Breathing Phantom the original lungs were replaced with a solid low-density foam which did not deform under pressure. An inflatable wedge bladder (IWB) was fitted between the solid foam and the phantom ribs to provide controlled chest motion. Finally, two silicone breast surrogates, laced with glass micro-beads to reduce the HU number were produced with a ball bearing (BB) located at the approximate centre. The breast surrogate displayed an average HU value of 6, showing good tissue equivalence, and the BB allowed fiducial identification during MV imaging. The surrogates were held on the phantom chest using a fine netting material, removed for the image in Fig. 1.

**Fig. 2:**
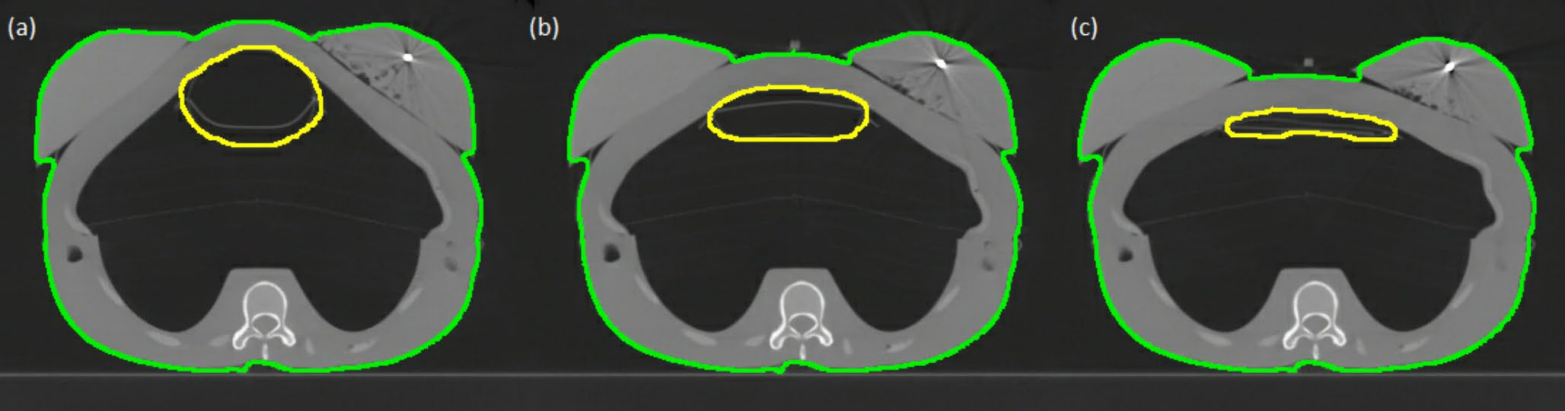
A CT image slice of the phantom in the (**a**) DIBH_H_ (**b**) DIBH_L_ and (**c**) exhale positions. The BB in the left breast is visible. The yellow contour delineates the position and volume of the IWB

A valve was fitted to the IWB pipe, external to the chest region. Two distinct settings released air at controlled rates. A constant air flow was delivered to the IWB by the Breathing Phantom air compressor to achieve a steady state volume of air in the IWB. The valve allowed two different DIBH positions to be achieved. CT images of the phantom in each volume configuration are shown in Fig. 2.

To verify the repeatability of the phantom DIBH position, multiple CT scans were completed. A scan was captured in the exhale (no air flow applied) position. A DIBH was then simulated with the air valve set to the low inhale volume (DIBH_L_). The air flow was ceased, the phantom was left for thirty seconds to return to the exhale position and another CT was captured. This process was repeated for a total of three cycles. A further three cycles of scanning were then captured with the air valve set to the high inhale volume (DIBH_H_). CT scans of each position, exhale, DIBH_L_ and DIBH_H_, were repeated on a second day to verify the variation between repeat set-ups. All CT images were captured using a GE Discovery RT scanner (General Electric, Boston, USA) and the clinical breast CT protocol, implementing 120 kV, automatic mAs optimisation, a CT slice thickness of 2.5 mm and in slice pixel size of 0.977 × 0.977 mm.

The external patient contour was defined for each scan in MIM. The created contours were compared against those from like scans using the Mean Distance to Agreement (MDA) and Dice Similarity Scores (DSC).

### Surface contour generation

During the prepositioning workflow the SLS system compares the patient position to a contour exported from the TPS. An optimum threshold value for generation of external contours should therefore be used to ensure the best possible match between the determination of the patient surface by the SLS and x-ray imaging.

Patient contours were created within MIM for each of the four phantoms with variable threshold HU values. Values of -600 HU, -400 HU, -200 HU and −100 HU were used. A 0 HU threshold resulted in visually incorrect determination of the patient external surface and was therefore not used. Two additional ‘Ultra Tight’ patient contours were created. These were the −200HU threshold contour contracted by 1 or 2 mm isotropically. Plans were exported to ETD for each contour when defined as the external contour.

The ETD x-ray system was used in combination with iGuide and the 6DoF HexaPod (Elekta AB, Stockholm, Sweden) system to position each phantom at isocentre in turn. Corrections were applied until all ETD x-ray reported corrections were < 0.2 mm & <0.1 degrees. The prepositioning module of ETD was then initiated for each HU threshold contour and the required shifts reported by the ETD pre-positioning were recorded as the difference between SLS and x-ray determined phantom position.

### X-ray imaging parameters

Optimal x-ray image settings are patient and observer dependent. The optimal images allow the automatic fusion software to run accurately, giving reliable corrections, while providing observers with high quality images which allow them to qualitatively assess the accuracy of the match and patient setup.

Two plans, one for each breast, were created with an isocentre located at approximately the centre of the breast. An exhale scan was used to remove uncertainties related to phantom inflation. The reference images were exported to the XVI v5.04 CBCT software (Elekta AB, Stockholm, Sweden) and ETD. CBCT imaging and 6DoF corrections were applied until residual errors were < 0.2 ± 0.1 mm & < 0.1 ± 0.1 degree. ETD images were then captured using variable kV and mA settings and the reported positional errors were recorded following the automatic fusion. For all images the optimal software filter was used for each image, based on user visual assessment. The fusion parameters were set to tissue and the spine and ball bearing were removed from the fusion. Three treatment radiation therapists were present for testing and provided qualitative feedback on visual quality. The ExacTrac, XVI and MV isocentres are routinely verified to differ by less than a maximum 0.5 mm in any cardinal direction.

### End to end testing

Repeat testing of the system in an E2E fashion was completed via hidden target testing. Treatment plans were created in Monaco v5.11 (Elekta AB, Stockholm, Sweden) consisting of 2 × 2 cm fields at Gantry 90 and 0 with the isocentre at the centre of the ball bearing inside the breast tissue. The phantom underwent the clinical workflow using the previously determined optimal imaging parameters and HU threshold value. On capture of the x-ray images the fusion was completed and the suggested corrections were applied using iGuide. Once in the final treatment position, a CBCT image was acquired along with MV portal images from the treatment plan fields. The centre of the BB position in each MV image was obtained relative to the field edges using an inhouse MATLAB (Mathworks, Natick, MA) script and corrected for inherent mechanical linac positioning variations using a full Winston Lutz test.

#### Standard workflow

E2E testing was completed three times for a single plan (DIBH_H_) and two times for a second plan (DIBH_L_). The mean positional error and standard deviation were calculated for each degree of freedom across the five tests.

#### Bone matching and tissue matching

The workflow testing was repeated for the DIBH_H_ while the fusion parameters were left at full bone match weighting during image matching. The BB and spine were manually excluded from the bone fusion, ensuring matching of the ribs rather than breast tissue.

#### Variation in breath hold volume

If a patient is unable to reproduce the expected change in chest elevation, for example due to an inability to inhale sufficient volume, the workflow will not proceed. If it is determined that there has been a change in the ability to achieve the DIBH position, the patient may be re-simulated, or the desired change in height of the tracking point may be manually overridden by the treating team.

During testing, the DIBH_H_ plan was loaded while the DIBH_L_ position was applied to the phantom, simulating an inadequate level of inspiration. The DIBH tracking point height was manually adjusted to match the value being achieved by the phantom and the workflow was continued to assess target positioning.

## Results

### Validation of phantom

Table 1 shows the MDA and DSC values obtained when comparing contours generated on multiple images of the breast phantom. The average and maximum or minimum values are shown for comparison of repeat scans on the same day and repeat scans across two days.

**Table 1:** The Mean Distance to Agreement (MDA) and Dice Similarity Coefficient (DSC) for contours generated on repeat imaging of the breast phantom

	MDA (mm)		DSC	
	Mean	Max	Mean	Min
Day 1 variation in exhale	0.31	0.53	0.996	0.994
Day 2 variation in exhale	0.08	0.12	0.999	0.999
Day 1 variation in DIBH_O_	0.15	0.17	0.998	0.998
Day 1 variation in DIBH_C_	0.14	0.18	0.999	0.998
Day 1 vs. Day 2 variation in exhale		0.36		0.996
Day 1 vs. Day 2 variation in DIBH_O_		0.35		0.996
Day 1 vs. Day 2 variation in DIBH_C_		0.35		0.996

The visual inter CT scan assessment found no observable differences in phantom position which would not be detected via contour metrics alone. These results indicated sufficient repeatability in phantom positioning for E2E testing.

### Surface contour generation

**Fig. 3:**
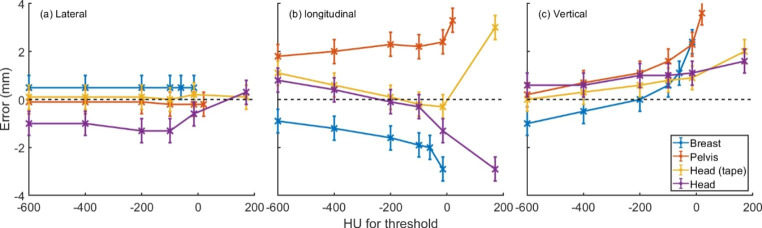
The reported pre-positioning shifts for a phantom in the correct location as defined by x-ray imaging. Error bars indicate the variability in the reported pre-positioning value over a 10 s period

The plots in Fig. 3 show the reported pre-positioning shifts against HU used for contour thresholding following positioning of the phantom using X-ray imaging. The Ultra tight threshold results are plotted against the average HU from a 1 mm rind of the associated contour.

It can be seen from the plots that no optimal value of HU threshold can be determined for all phantoms. This is likely due to the different materials and colours of the phantoms and the subsequent changes in their behaviour under CT and SLS imaging. The Ultra tight contours produced inaccurate positioning and should not be used. HU values between −600 and −200 HU provided adequate results and showed changes of less than 1 mm in reported position across the range of contours.

### X-ray imaging parameters

**Fig. 4:**
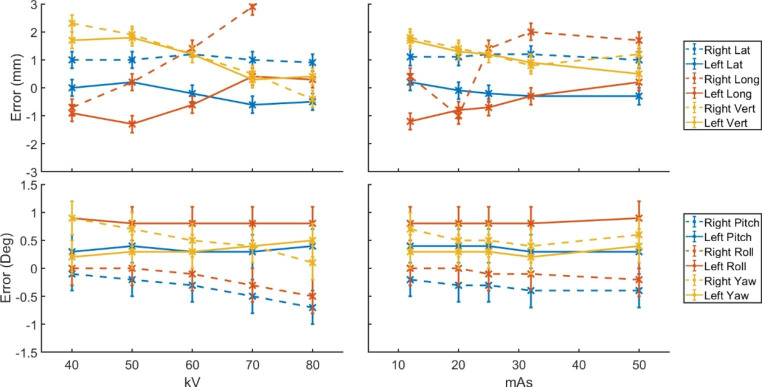
The reported error in position (mm or degrees) against x-ray potential (kV) or current (mAs) following CBCT placement

The plots in Fig. 4 show the x-ray reported errors for varied kV and mA when imaging a phantom positioned using CBCT. For variable kV settings, 25mAs was maintained, for variable mAs a fixed 60 kV was maintained.

No combination of settings determined zero errors in all directions. This is likely due to differences in fusion algorithm, and slight variations in isocentre calibration between XVI and ETD (< 0.5 mm). Tube voltages of 50-60 kV provided results which did not differ by more than 2 mm or 1 degree from the CBCT suggested position. The radiation therapists reported optimal visual contrast for voltages between 50 and 70 kV and mAs values between 20 and 32 mAs.

### End to end testing

#### Standard workflow

**Fig. 5:**
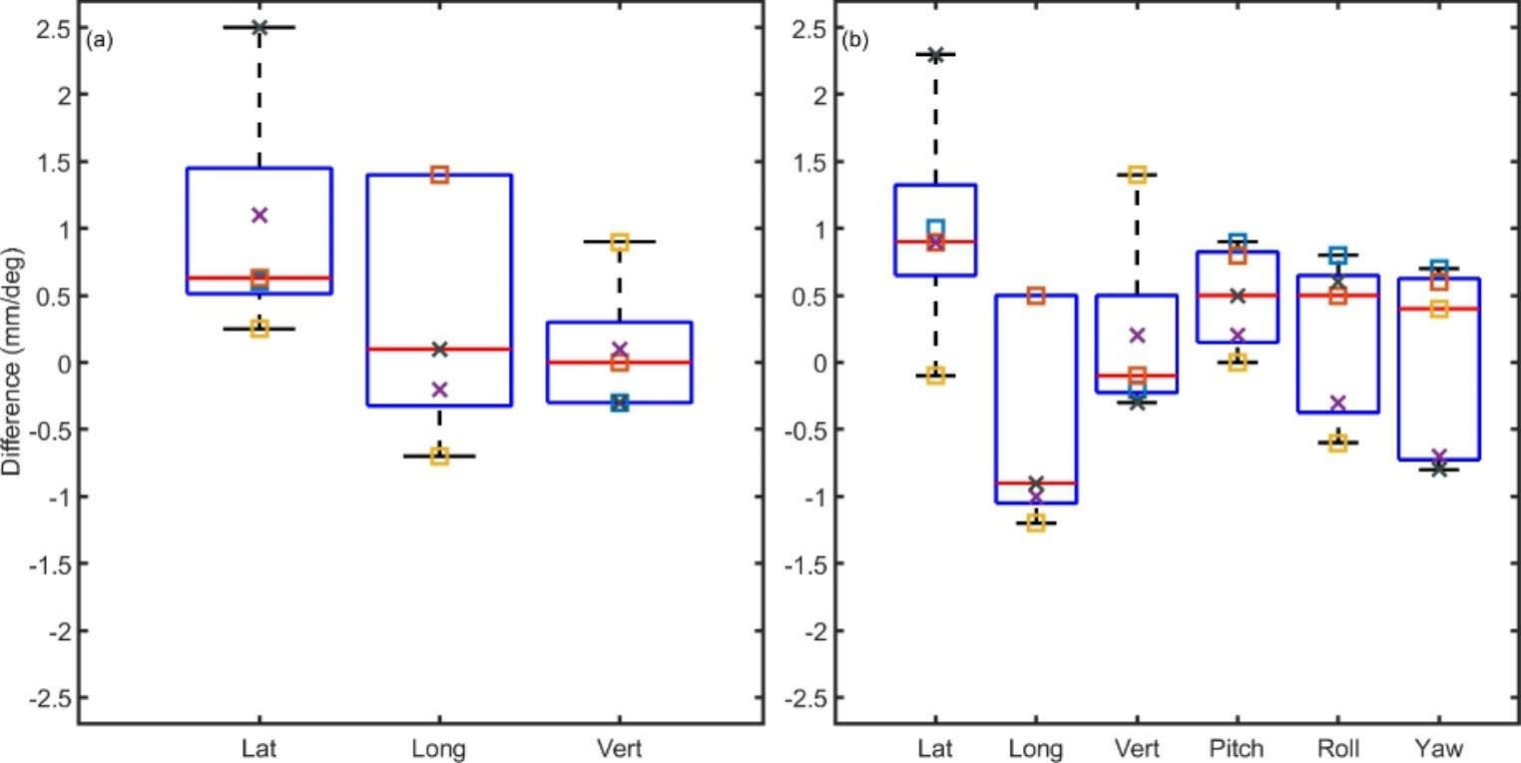
The residual error in phantom position as measured by (**a**) distance of the ball bearing position from MV isocentre and (**b**) CBCT fusion. The same colour markers indicate the same E2E test, the same shape indicate the same E2E plan

A total of five E2E tests, using two plans, were completed. Figure 5 summarises the residual positioning error measured via deviation of a BB from isocentre and CBCT, following completion of the E2E workflow. The central marks on the boxplots display the median value, and the edges of each box show the 25th and 75th percentiles (the interquartile range (IQR)).

Over the five end to end tests the average deviation of the BB from the MV isocentre was 1.0 mm, 0.4 mm and 0.1 mm in the lateral, longitudinal and vertical directions respectively. The corresponding standard deviations were 0.9 mm, 1.0 mm and 0.5 mm. The average residual error in the CBCT images were −0.6 mm, 0.5 mm and 0.2 mm in the lateral, longitudinal and vertical directions, with standard deviations of 1.1 mm, 0.7 mm and 0.4 mm respectively. The rotational errors showed average values of 0.0^o^, 0.5^o^ and −0.8^o^ with standard deviations of 1.2^o^, 1.7^o^ and 1.8^o^ for pitch roll and yaw respectively.

#### Bone matching and tissue matching

Bone matching settings within the fusion process resulted in increased residual error in the phantom position compared to tissue matching. The measured errors are displayed in Table 2.

**Table 2:** The measured errors in final phantom position via CBCT imaging and MV imaging when using bone weighted image matching

Direction	CBCT calculated error	MV calculated error
Lateral (mm)	2.1	2.1
Longitudinal (mm)	5.3	-5.1
Vertical (mm)	-0.9	-0.9
Pitch (deg)	1.2	
Roll (deg)	0.1	
Yaw (deg)	-1.5	

These results indicate that the positional errors were over 5 mm in the longitudinal direction and the rotational errors exceed 1.5^o^.

#### Variation in breath hold volume

To allow the workflow to continue with the phantom in the DIBH_L_ configuration, when expecting a DIBH_C_ configuration, the tracking point height was manually adjusted from the ETD calculated value of 37.3 mm to 19.6 mm. Following the completion of the imaging and positional corrections the errors in the phantom position were as recorded in Table 3.

**Table 3:** The measured errors in final phantom position via CBCT imaging and MV imaging following a manual reduction of the DIBH level

Direction	CBCT calculated error	MV calculated error
Lateral (mm)	-1.0	0.3
Longitudinal (mm)	-0.8	-0.2
Vertical (mm)	-0.3	-0.3
Pitch (deg)	-1.5	
Roll (deg)	-4.0	
Yaw (deg)	-4.0	

Large errors in rotations were reported following this process, likely due to the change in phantom shape with reduced inhale volume. Particularly, the reduced IWB volume reduced the roll of the breast position from the midline. The translational errors were all observed to be sub mm, indicating that the centre of the target was located at isocentre following the compromised fusion ability.

## Discussion

From the results of the testing completed in this study, a range of HU values suitable for external contour generation were identified along with x-ray imaging parameters suitable for breast imaging using the stereoscopic x-ray system. The workflow was tested under a range of clinical scenarios and found to position the phantom correctly with an accuracy inside the TG302 recommended values for DIBH breast treatments of 3-5 mm and 2-3^O^ [5].

The use of a phantom with breathing motion provides a more clinically representative demonstration of the workflow, however it also introduces a higher level of uncertainty into the measurement process. The phantom was shown to be able to accurately achieve the same position over time and variations in position were well below the 2 mm tolerance applied during the workflow. Similar studies of the ETD system have often considered use in stereotactic radiotherapy when smaller treatment margins demand higher positional accuracy [14, 15]. They have shown performance of the system against static phantoms to be equal to or greater than the repeatability of the phantom used in this work. This should be considered against the results of this study, and the required accuracy for clinical DIBH.

Prepositioning the patient using the SLS system has two primary functions; minimising IGRT residual positioning errors to allow corrections without large movements of the patient and ensuring the BH level of the day is calculated correctly. If the SLS system identification of the patient differs from the CT generated contours, the BH level of the day will be calculated at the wrong position in relation to the FB and BH contours. Differences were observed in the prepositioning of the phantom depending on the HU threshold used for contouring. For a wide HU range of -600 to -200HU, these differences varied by less than 1 mm across the HU range, and the errors remained under 2.5 mm, equivalent to 1 slice thickness of the reference CT. In clinical practice, a patient would additionally be free breathing during this prepositioning process introducing natural variations in the chest height in the range of 3 mm [5]. These results indicate that the phantom was positioned with sufficient accuracy to ensure both minimal subsequent moves would be required and the BH level of the day would be calculated at the correct slice of the CT.

Due to the variability in individual patient characteristics, optimal imaging parameters are patient specific. Interobserver variations can also result in different fusion, or fusion assessment, outcomes. In clinical practice independent assessment of each automatic fusion should be made by the treating team and algorithm results should not be trusted blindly. This process was not applied to the results presented in Fig. 4 to determine if those images showing larger residual errors related to poorer image fusion by the algorithm around the breast tissue. Lower kV values of 50–60 kV did show optimal automated fusion results. This is attributed to the increased contrast in the breast tissue which was believed to increase the weighting of the fusion algorithm towards the breast tissue and away from the bone anatomy which was not as clearly visible as when using high kV values. During the initial clinical release of the system 60 kV and 25 mAs will be the default imaging parameters within our department, however they are to be reviewed by the treatment team in relation to the specific patient.

CBCT image results were used in this study as an independent review of ETD due to the common use of this IGRT modality within the department. Settings and practices when using the XVI software can result in varied image fusion results. The area of focus during the XVI fusion is user control via definition of a clip box on the reference image. The box must however be rectangular, ensuring sections of the chest wall and lung were mandatory when including the breast tissue. The ETD software enables identification of areas to be excluded from the fusion algorithm by free hand illustration, in addition to the bone and tissue weightings being variable, again resulting in different fusion results for the same captured images. The comparison of ETD and XVI reported residual errors can therefore inform of difference in expected position between systems and fusion algorithms but cannot indicate which is inherently more accurate [15].

During the routine workflow hidden target tests the residual error in phantom position was observed to be within 2.5 mm and 1^o^. The phantom was therefore positioned with an accuracy approximately equal to one CT slice thickness which is expected to be sufficient for DIBH breast workflows [5].

When using a bone weighted fusion, as is typical during cranial treatments, the final phantom position showed greater errors than those during the five tissue weighted fusion hidden target tests. Most notable was the error greater than 5 mm in the longitudinal direction. During the assessment of the image fusion, it was visually apparent that the breast volume was not optimally matched. It is possible the quality of the fusion was reduced due to the diminished contrast of the bony components of the phantom when using lower kV imaging which is more suited to assessing soft tissue. This would likely have been adjusted if observed during a clinical workflow, however it is suggested the tissue weighting be given a strong preference when treating primarily soft tissue targets. The clinical team should consider the relevance of this result for post mastectomy patients or those with reduced soft tissue mass.

During the simulated reduction in BH volume the target volume was aligned well to the isocentre, indicating potential for the treatment to progress. The XVI reported errors identified the significant change in phantom shape due to the reduced breath hold. This would likely affect the treatment target coverage and OAR sparing relative to the planned dosimetry. Care should therefore be taken in reducing the breath hold level of the day to account for patient change. It may be suitable to set a limit on the magnitude of the change that is considered acceptable or the number of fractions for which a reduced volume could be tolerated. If CBCT imaging is possible within the department, it could provide additional insight to potential changes to the heart dose during these cases and aid in the decision with respect to delivering treatment on that day.

## Conclusion

A series of tests were completed to evaluate the ETD DIBH breast workflow in an E2E fashion using hidden target testing. It was shown that a broad range of HU values can be used to create external contours for the preposition workflow without large variations in the final positioning of the patient. This minimises the risk of the prepositioning workflow locating the patient incorrectly and subsequently calculating an incorrect height change for the tracking point. Optimal fusion results and visual assessments of stereoscopic images were reported with lower kV values in the range of 50–60 kV due to increased soft tissue contrast. Default initial x-ray parameters were established for our department of 60 kV and 25mAs, to be varied if required depending upon the individual patient. During hidden target testing the phantom was positioned within 2.5 mm and 1^o^ of the expected position during five different instances and good agreement was observed between the ETD and XVI image fusions. Reduced accuracy was observed in phantom positioning when bone weighting was used for image fusion in place of tissue weighting but isocentre positioning was maintained when the breath hold level of the day was manually reduced in response to a simulated reduced breath hold capacity despite changes in phantom geometry. This offers the clinical team the decision to deliver the optimum treatment of the day or to delay until better positioning or re-simulation can be achieved. The results of this initial testing indicate that the ExacTrac Dynamic breast DIBH workflow is able to position a phantom within the TG302 recommended tolerances [5].

## References

[1] Simonetto C, Eidemüller M, Gaasch A (2019). Does deep inspiration breath-hold prolong life? Individual risk estimates of ischaemic heart disease after breast cancer radiotherapy. Radiother Oncol.

[2] Bergom C, Currey A, Desai N, Tai A, Strauss JB (2018). Deep inspiration breath hold: techniques and advantages for Cardiac Sparing during breast Cancer irradiation. Front Oncol.

[3] Haussmann J, Corradini S, Nestle-Kraemling C (2020). Recent advances in radiotherapy of breast cancer. Radiation Oncol (London England).

[4] Smyth LM, Knight KA, Aarons YK, Wasiak J (2015). The cardiac dose-sparing benefits of deep inspiration breath‐hold in left breast irradiation: a systematic review. J Med Radiat Sci.

[5] Al-Hallaq HA, Cerviño L, Gutierrez AN (2022). AAPM task group report 302: surface‐guided radiotherapy. Med Phys (Lancaster).

[6] Kalet AM, Cao N, Smith WP (2019). Accuracy and stability of deep inspiration breath hold in gated breast radiotherapy – A comparison of two tracking and guidance systems. Physica Med.

[7] Li G, Lu W, O’Grady K (2022). A uniform and versatile surface-guided radiotherapy procedure and workflow for high‐quality breast deep‐inspiration breath‐hold treatment in a multi‐center institution. J Appl Clin Med Phys.

[8] Laaksomaa M, Sarudis S, Rossi M (2019). AlignRT® and Catalyst™ in whole-breast radiotherapy with DIBH: is IGRT still needed?. J Appl Clin Med Phys.

[9] Freislederer P, Batista V, Öllers M (2022). ESTRO-ACROP guideline on surface guided radiation therapy. Radiother Oncol.

[10] Rong Y, Walston S, Welliver MX, Chakravarti A, Quick AM (2014). Improving intra-fractional target position accuracy using a 3D surface surrogate for left breast irradiation using the respiratory-gated deep-inspiration breath-hold technique. PLoS ONE.

[11] Alderliesten TP, Sonke J-JP, Betgen AM, Honnef JM, van Vliet-Vroegindeweij CP, Remeijer PP (2013). Accuracy evaluation of a 3-Dimensional Surface Imaging System for Guidance in Deep-Inspiration breath-hold Radiation Therapy. Int J Radiat Oncol Biol Phys.

[12] Nankali S, Hansen R, Worm E (2022). Accuracy and potential improvements of surface-guided breast cancer radiotherapy in deep inspiration breath-hold with daily image-guidance. Phys Med Biol.

[13] Crop F, Laffarguette J, Achag I (2023). Evaluation of surface image guidance and deep inspiration breath hold technique for breast treatments with Halcyon. Physica Med.

[14] Chow VUY, Cheung MLM, Kan MWK, Chan ATC (2022). Shift detection discrepancy between ExacTrac dynamic system and cone-beam computed tomography. J Appl Clin Med Phys.

[15] Da Silva Mendes V, Reiner M, Huang L (2022). ExacTrac dynamic workflow evaluation: combined surface optical/thermal imaging and X-ray positioning. J Appl Clin Med Phys.

[16] Astudillo R, Gutierrez Ruiz M, Alonso Muriedas J (2021). PO-1556 evaluation of the new Brainlab Exactrac dynamic structured light positioning system. Radiother Oncol.

[17] Li J, Shi W, Andrews D (2017). Comparison of online 6 degree-of-Freedom Image Registration of Varian TrueBeam Cone-Beam CT and BrainLab ExacTrac X-Ray for intracranial radiosurgery. Technol Cancer Res Treat.

[18] Ma J, Chang Z, Wang Z, Jackie Wu Q, Kirkpatrick JP, Yin F-F (2009). ExacTrac X-ray 6 degree-of-freedom image-guidance for intracranial non-invasive stereotactic radiotherapy: comparison with kilo-voltage cone-beam CT. Radiother Oncol.

[19] Batumalai V, Phan P, Choong C, Holloway L, Delaney GP (2016). Comparison of setup accuracy of three different image assessment methods for tangential breast radiotherapy. J Med Radiat Sci.

[20] Bright M, Foster RD, Hampton CJ, Ruiz J, Moeller B (2022). Failure modes and effects analysis for surface-guided DIBH breast radiotherapy. J Appl Clin Med Phys.

